# Measurement Properties of the Staff Attitude to Coercion Scale: A Systematic Review

**DOI:** 10.3389/fpsyt.2022.744661

**Published:** 2022-04-28

**Authors:** Tonje Lossius Husum, Torleif Ruud, Jakub Lickiewicz, Johan Siqveland

**Affiliations:** ^1^Centre for Medical Ethics, University of Oslo, Oslo, Norway; ^2^Faculty of Health Sciences, Oslo Metropolitan University, Oslo, Norway; ^3^Mental Health Services, Akershus University Hospital, Lørenskog, Norway; ^4^Institute of Clinical Medicine, University of Oslo, Oslo, Norway; ^5^Department of Health Psychology, Jagiellonian University Medical College, Krakow, Poland; ^6^National Centre for Suicide Research and Prevention, Institute of Clinical Medicine, University of Oslo, Oslo, Norway

**Keywords:** mental health, staff, attitudes, coercion, psychometrics, systematic review

## Abstract

**Objective:**

The Staff Attitude to Coercion Scale (SACS) was developed to assess mental health care staff's attitudes to the use of coercion in treatment. The staff's attitudes to the use of coercion may also influence their willingness to engage in professional development projects aimed at reducing use of coercion. This study systematically reviews the existing evidence related to the measurement properties of the SACS in papers published since the publication of SACS in 2008.

**Methods:**

Seven databases were searched for studies published until October 2021 assessing the measurement properties of SACS or using SACS. All original studies reporting data relevant for the assessment of measurement properties of the SACS were eligible for inclusion. The methodological quality of the studies was assessed and rated using the COnsensus-based Standard for the selection of health Measurement INstruments (COSMIN).

**Results:**

Of the 81 identified publications, 13 studies with a total of 2,675 respondents met the inclusion criteria. Most studies reported data on structural validity and internal consistency, with high methodological quality, but there were almost no data on any other measurement properties.

**Conclusion:**

We found evidence for adequate structural validity and internal consistency of the SACS, while other important measurement properties were not addressed in any of the reviewed studies. Caution is needed when interpreting results of the SACS in terms of aspects such as reliability, criterion validity and measurement error. The relationship between staff attitudes to coercion and the actual use of coercion also remains unclear and needs to be further investigated.

**Systematic Review Registration:**

https://www.crd.york.ac.uk/prospero/, identifier: CRD42021239284.

## Introduction

The use of coercion in health care is ethically problematic and challenge the fundamental health care principle of respect for patient autonomy (1, 2). All over the world there are initiatives to minimize its use (3–6). Health care professionals need to critically reflect upon and morally justify each use of coercive interventions (7, 8). Several studies have shown considerable variation in use of coercive measures both, in one country and between different countries (9–11). These differences are not yet fully explained (12–14). Based on differences in the use of coercive practices among different countries, regions, and hospitals, some of the variation can be attributed to differences in staff attitudes to the use of coercion (15). Attitudes can be defined as a psychological tendency that is expressed through evaluating an entity with a normative degree of either positivity or negativity, based on experience (16). Attitudes do influence behavior, but the connection between attitudes and behavior is complex, and may depend on situational factors. The connection has not been fully mapped yet, and the relationship may also depend on the subject of the attitude (17).

In recent years, attitudes to the use of coercive interventions in mental health care have evolved, with increased focus on user participation, respect for autonomy, and human rights (6). Differences in staff attitudes to the use of coercion may explain why some wards and hospitals have attempted to reduce the use of coercion, while others have not made the same effort (4, 18). Staff attitudes to coercion may also influence the amount of coercion used and reveal the reasons for using coercion in treatment and the dynamics involved.

It is therefore important to have a validated questionnaire for assessing staff attitudes to coercion in mental health care. In 2008, Husum et al. developed and published the Staff Attitude toward Coercion Scale (SACS) for this purpose (19). SACS measures staff attitudes toward use of coercive practices in mental health care. SACS was developed as a short 15-item questionnaire with normative attitudes toward use of coercion. It consists of statements about the use of coercion, about how the participant thinks about it, and whether the participant considers coercive interventions necessary. Using factor analysis, the questionnaire divided staff attitudes to coercion into three groups: the view that use of coercion may offend (critical attitude); the view that coercion is necessary for care and security reasons (pragmatic attitude); and the view of coercion as a valid form of treatment (positive attitude). These items are scored on a five-point Likert scale, from 1 = strongly disagree to 5 = strongly agree.

The SACS is to our knowledge the only instrument measuring staff attitudes to coercion, and the instrument has now been used worldwide and translated into several languages, including German, Polish, Chinese, Japanese, Italian, Turkish and Arabic, indicating a potential for cross-cultural applicability. The questionnaire has also been used in some populations with other participants, like patients and caregivers. However, to the best of our knowledge, no systematic review has been performed to assess the measurement properties of the scale. To date, there has been no attempt to examine the results of its use in practice and research, and no meta-analysis has been done.

This review aims to gather results relevant for measurement evaluation of the SACS questionnaire. Assessing measurement characteristics is essential for comparing results from different countries and populations. In particular, the aim of this study was to conduct a systematic review of the measurement properties of SACS using the COSMIN Risk of Bias checklist (20–23). The following review questions were considered.

(1) Summarize and evaluate the available evidence regarding the measurement properties and use of the Staff Attitude to Coercion Scale (SACS) in health care settings.
(2) Assess the reliability and validity of the SACS as reported in these studies.
(3) Examine the performance and factorial invariance of the three SACS dimension ratings across subgroups [e.g., defined as populations from different countries; different professional groups; differences between other populations (patients and carers) and across time].

## Methods

This systematic review was carried out following the “COnsensus-based Standards for the selection of health Measurement Instruments” (COSMIN) (20, 22, 23) and the “Preferred Reporting Items for Systematic reviews and Meta-Analyses” (PRISMA) guidelines (24).

The review protocol was registered with PROSPERO (Record ID = CRD42021239284).

### Criteria for Selecting Studies

All original studies reporting data relevant for the assessment of measurement properties of the SACS were eligible for inclusion. There were no restrictions on setting or publication language. The systematic review includes studies reporting data from any eligible SACS measures on one or more of the domains defined by the consensus-based standard for the selection of health measurement instruments (COSMIN) taxonomy: reliability (internal consistency, test-retest reliability, inter-rater reliability, and measurement error); validity (content validity, construct validity, cross-cultural validity, predictive validity, criterion validity, and structural validity); responsiveness; and interpretability (20, 22).

### Strategies for the Identification of Studies

All relevant studies that met the inclusion criteria were identified by searching the following seven electronic databases: MEDLINE by EBSCOhost, PsycINFO by APA PsycNET, Embase by Elsevier, CINAHL by EBSCOhost, Web of Science by Thomson Reuters, Google Scholar, and OpenGrey. The following terms were used in the search for studies: SACS, Staff attitude to coercion scale, Staff attitude toward coercion scale, Staff's normative attitudes toward coercion. Studies published between 2008 and October 2021 were considered. The full search string is attached as Appendix 1. A trained librarian at the hospital library conducted the search. In addition, researchers who had asked for permission to use the scale were also contacted and asked for their results.

### Study Screening and Selection

**Figure 1:**
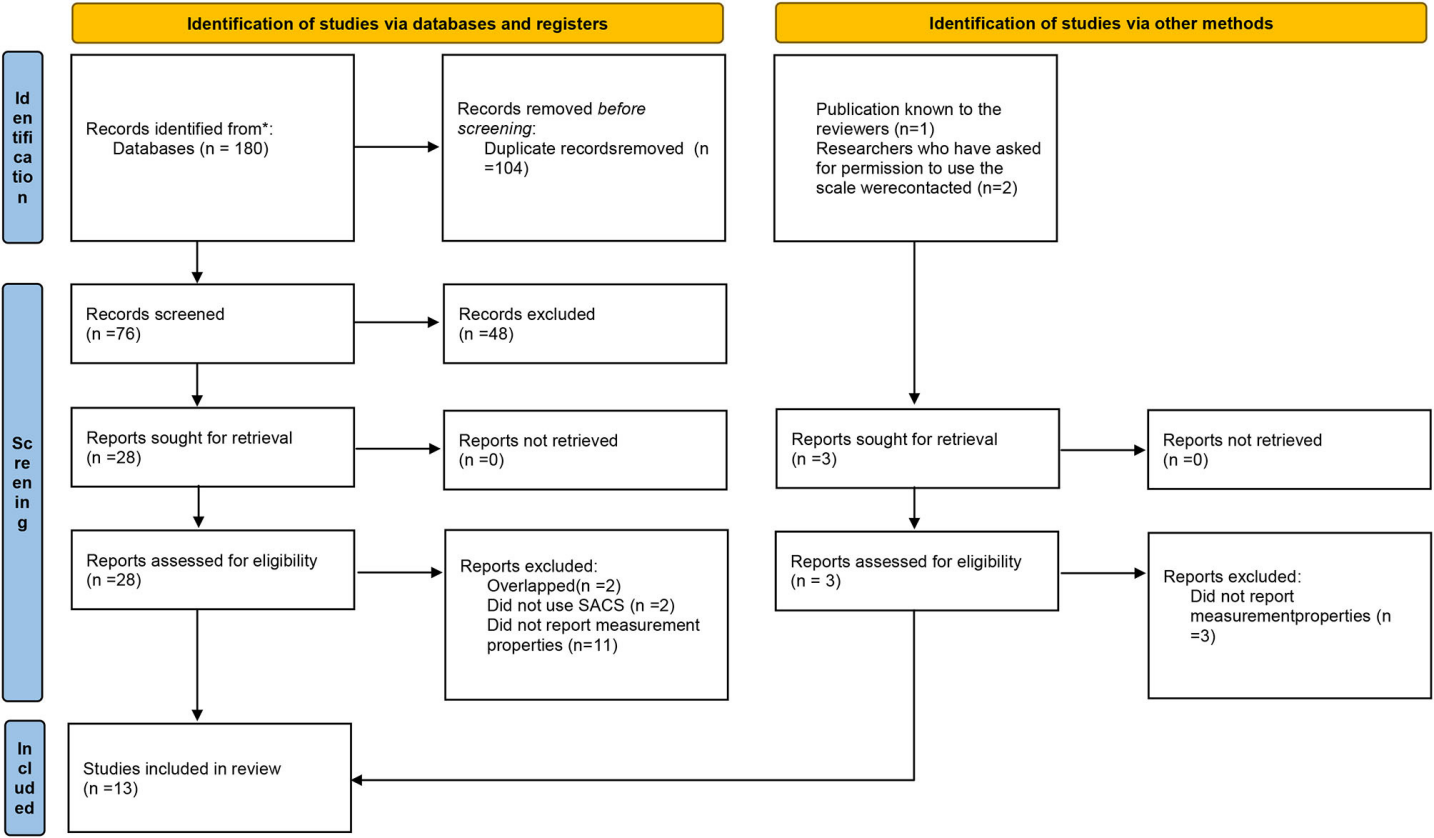
PRISMA 2020 flow diagram for new systematic reviews which included searches of databases, registers and other sources. ^*^Number of records identified from each database is presented in the Appendix 1 in the description of the database search. From: Page et al. (24). For more information, visit: http://www.prisma-statement.org/.

The selection of studies was made by two review teams (JL with TLH, and JS with TLH). Each review team reviewed half of the articles. A third reviewer (TR) was involved when there was discrepancy between the two reviewers. Any discrepancies regarding selection were resolved by consensus. First, titles and abstracts were screened for eligibility. Then, the full text of the potentially relevant studies was read to decide whether the study met the selection criteria. Studies that did not fulfill all the inclusion criteria were excluded, and the reason for exclusion was noted. A flow chart of the selection process is presented in Figure 1.

### Assessing the Risk of Bias

For assessing the measurement properties of SACS we used the COSMIN Risk of Bias checklist (21, 22). In their 10 steps for conducting a systematic review of patient-reported outcome measurements the COSMIN group defines the evaluation of measurement properties in three steps [step five to seven in Figure 1 in (22)] using the COSMIN Risk of Bias checklist: evaluate content validity, evaluate internal structure (structural validity, internal consistency, cross-cultural validity/measurement invariance), and evaluate the remaining instrument properties (reliability, measurement error, criterion validity, hypothesis testing for construct validity, responsiveness) (21, 22).

Using the checklist, two reviewers independently assessed the methodological quality of measurement properties reported in each study with discrepancies in assessment resolved by consensus. For this review of a single instrument, already developed, we did not rate the studies on content validity. Each of the eight other measurement properties were rated on a four-point scale (inadequate, doubtful, adequate, very good) according to the definitions and instructions in the COSMIN manual (20, 22). The rating inadequate is also used when a study has not examined or reported a property when this could have been done for the instrument.

### Data Extraction and Analyses

Two reviewers (JL and JS) extracted the data, and a third reviewer checked the data being extracted (TLH). Data related to internal consistency (Cronbach's alpha), structural validity (factor analysis, correlations), reliability (ICC, Cohen's kappa), and responsiveness (correlations) were collected. In cases of uncertainty about the extracted data, another reviewer (TR) was consulted. For further definition of the measurement constructs, please refer to the COSMIN manual.

## Results

### Study Selection

Our search resulted in 81 hits, which were reviewed at the title and abstract level. Altogether, 31 studies were read in full text. Finally, 13 studies were included, while the rest were excluded, with reasons for exclusion at the full text level given in Figure 1.

### Study Population Demographics

The 13 included studies had between 39 (25) and 424 participants (26), with a total of 2,675 respondents. The studies were from eight different countries and used six different language versions. All populations included mental health professionals, either mixed or grouped by profession. The only exception was one population of 210 caregivers (27) (Table 1).

**Table 1:** General characteristics of the included studies.

**References**	**Country**	**Population (*n*)**	Setting*	**Response rate**	**Measurement properties reported**
Arab et al. (28)	Iran	Physicians, nurses, and paramedics (273)	PH	91%	Structural validity, internal consistency
Efkemann et al. (29)	Germany	Mental health professionals (209)	PH	No data	Structural validity, internal consistency
Elmer et al. (30)	Switzerland/Germany	Mental health professionals (424)	PH/MS	26%	Internal consistency, hypothesis testing
Husum et al. (19)	Norway	Multidisiplinary staff groups (215)	PH	No data	Structural validity, internal consistency
Kiejna et al. (30)	Poland	Multidisiplinary staff groups (120)	PH	No data	Structural validity, internal consistency, reliability
Krieger et al. (30)	Germany	Multidisiplinary staff groups (138)	PH	13.8%	Internal consistency
Lambert et al. (31)	UK	Nursing staff (63)	PH	No data	Structural validity
Molewijk et al. (8)	Norway	Multidisiplinary staff groups (379)	PH	No data	Internal consistency
Motteli et al. (32)	Switzerland	Multidisiplinary staff groups (110)	PH	36%	Internal consistency
Orlick (33)	USA	Nursing staff/patient care technicians (50)	PH	73.5%	Internal consistency
Rabenschlag et al. (25)	Switzerland	Staff (39)	PH	49%	Internal consistency
Raveesh et al. (27)	India	Psychiatrists (210) and caregivers (210)	PH	No data	Internal consistency
Wu et al. (34)	Taiwan	Psychiatric social workers (235)	PH	59%	Internal consistency

* *Setting: PH, psychiatric hospitals; MS, medical students*.

### Structural Validity

We identified five studies assessing structural validity (19, 28–31). Four of these studies reported data from factor analysis (19, 28–30), while one reported correlations between the subscales (31).

Husum et al. (19) was the first study in this category and reported three factors representing the underlying theoretical structure and explaining 49.1% of the variance. This finding was later replicated (28, 30). Arab et al. found that the three factors explained 61.93% of the variance, while Kiejna et al. found that they explained 52.3% of the variance.

Efkemann et al. reported four factors meeting the common factor analysis criteria of eigenvalue <1; however, the last factor was only marginally larger than one. A scree plot inspection indicated that a three-factor solution might better represent the underlying structure. When testing the original three-factor solution reported by Husum et al. (19), however, they found that not all items corresponded to the original factor solution, and that some items loaded on two factors. As a final test of the internal structure, they did a factor analysis with only one factor. This also seemed to represent an adequate solution, as all items loaded higher than 0.4 on this factor.

The study reporting subscale correlations found that the “coercion as offending” subscale correlated to 0.34 with the “coercion as care and security” subscale and to −0.12 with the “coercion as treatment” subscale. The “coercion as care and security” and the “coercion as treatment” subscales correlated to 0.65 (31) (Table 2).

**Table 2:** Structural validity and internal consistency reported by studies.

**References**	**Factors**	**Explained variance**	**Internal consistency (Cronbach's alpha) of scale**			
			**Total scale**	**Coercion as offending**	**Coercion as care and security**	**Coercion as treatment**
Arab et al. (28)	3	61.93%	0.71	0.72	0.89	0.76
Efkemann et al. (29)	1	-	0.84	0.76	0.76	0.76
Elmer et al. (26)	3	n/a	-	0.61	0.63	0.71
Husum et al. (19)	3	49%	0.78	0.69	0.70	0.73
Kiejna et al. (30)	3	52.3%	0.82	0.74	0.81	0.57
Krieger et al. (30)*	3	-	0.83	**	**	**
Molewijk et al. (8)	3	-	-	0.67	0.71	0.67
Motteli et al. (32)	3	-	-	0.69	0.77	0.69
Orlick (33)	3	-	Pre:0.84 Post:0.84	Pre:0.70 Post:0.67	Pre:0.92 Post:0.90	Pre:0.80 Post:0.75
Rabenschlag et al. (25)	3	-	0.65	<0.60	<0.60	<0.60
Raveesh et al. (27)	3	-	0.58	0.44	0.69	0.57
Wu et al. (34)	3	-	0.68	-	-	-

* *Used a 4-point scale (instead of 5-point)*.

** *Not applicable*.

The studies investigating structural validity were overall of good methodological quality; two studies were rated very good and one adequate, fair (Table 3).

**Table 3:** Methodological quality^*^ of the studies by measurement property.

**References**	**Structural validity**	**Internal consistency**	**Measurement invariance**	**Reliability (test-retest)**	**Measurement error**	**Criterion validity (compared to gold standard)**	**Hypothesis testing**	**Responsiveness (sensitivity to change)**
Arab et al. (28)	Doubtful	Very good	Inadequate	Inadequate	Inadequate	Inadequate	Inadequate	Inadequate
Efkemann et al. (29)	Adequate	Very good	Inadequate	Inadequate	Inadequate	Inadequate	Inadequate	Inadequate
Elmer et al. (26)	Inadequate	Very good	Inadequate	Inadequate	Inadequate	Inadequate	Adequate	Inadequate
Husum et al. (19)	Very good	Very good	Inadequate	Inadequate	Inadequate	Inadequate	Inadequate	Inadequate
Kiejna et al. (30)	Very good	Very good	Inadequate	Doubtful	Inadequate	Inadequate	Inadequate	Inadequate
Krieger et al. (30)	Inadequate	Adequate	Inadequate	Inadequate	Inadequate	Inadequate	Inadequate	Inadequate
Lambert et al. (31)	Inadequate	Inadequate	Inadequate	Inadequate	Inadequate	Inadequate	Inadequate	Inadequate
Molewijk et al. (8)	Inadequate	Very good	Inadequate	Inadequate	Inadequate	Inadequate	Inadequate	Inadequate
Motteli et al. (32)	Inadequate	Very good	Inadequate	Inadequate	Inadequate	Inadequate	Inadequate	Inadequate
Orlick (33)	Inadequate	Doubtful	Inadequate	Inadequate	Inadequate	Inadequate	Inadequate	Inadequate
Rabenschlag et al. (25)	Inadequate	Doubtful	Inadequate	Inadequate	Inadequate	Inadequate	Inadequate	Inadequate
Raveesh et al. (27)	Inadequate	Very good	Inadequate	Inadequate	Inadequate	Inadequate	Inadequate	Inadequate
Wu et al. (34)	Inadequate	Doubtful	Inadequate	Inadequate	Inadequate	Inadequate	Inadequate	Inadequate

* *Methodological quality reported with the four level ratings of COSMIN Risk of Bias Checklist: Very good, adequate, doubtful, inadequate*.

### Internal Consistency

This was the most frequently reported measurement property, with 12 studies reporting relevant analysis (8, 19, 25–30, 32–35). Nine studies reported Cronbach's alpha for the entire SACS scale, varying between 0.58 (27) and 0.84 (29, 33). Six of the nine studies (19, 25, 27–30, 33–35) reported alpha above 0.70. Nine studies reported Cronbach's alpha for the “coercion as offending” subscale, varying between 0.44 (27) and 0.76 (29). Four studies reported Cronbach's alpha at or above 0.70 (28–30, 33). Cronbach's alpha for the “coercion as care and security” subscale was reported in nine studies, and varied between 0.63 (26) and 0.89 (28). Seven studies reported Cronbach's alpha at or above 0.7 (8, 19, 25, 27–29, 32–35). Cronbach's alpha for the “coercion as treatment” subscale was reported in nine studies, and varied between 0.57 (27) and 0.80 (33). Five studies reported Cronbach's alpha at or above 0.70 (19, 26, 28, 29, 33) (Table 2). The methodological quality of reporting internal consistency was very good for eight studies, adequate for one, doubtful for three and inadequate for one (Table 3).

### Other Measurement Properties

Only two studies reported data on other measurement properties reported in Table 3; Kiejna et al. (30) reported test-retest reliability over 3 weeks and found a correlation of 0.57 between the time points. Elmer et al. (26) investigated the relationship between SACS and attitudes to informal coercion among medical students and health care personnel. They found positive attitudes toward coercion to be negatively associated with recognizing informal coercion. On the other side, personnel viewing coercion as offending recognized coercion more adequately.

## Discussion

The SACS has been used in many studies and countries since its development in 2008. This indicates concern in many cultural settings about use of coercion in mental health care. However, to the best of our knowledge, this is the first systematic review of the measurement properties of the SACS.

Structural validity and internal consistency were the most frequently reported measurement characteristics in the identified studies. All studies reported adequate internal consistency. Most studies replicated the original three-factor structure and the correlation between the factors were medium to low as expected. Merging the model into one factor has been considered, but the measurement findings in this review suggest that the three-factor is better fit to the available data. Keeping the three-factor model is supported by another study who found that professionals could be divided in three groups concerning their thoughts about use of seclusion in mental health care. The authors of this study identified three types of professionals: Transformers, Doubters, and Maintainers (36).

While the available data support the SACS as being psychometrically sound, some important data are missing. For example, there are no measurements across more extended periods of time (37), which could be used to assess the sensitivity to change and stability of the SACS. Two test-retests within a 3-week interval indicate that SACS score may vary over time in the same individuals indicating that the SACS may assess dynamic attitudes likely to fluctuate even within relative short time periods.

Testing of criterion validity by comparing SACS to a gold standard is lacking because there are no other instruments on staff attitude to coercion to compare with. There is also very limited research on the relationship between staff attitudes and the use of coercion. Several studies have suggested that there is a relationship between staff attitudes toward use of coercion and actual use of coercive interventions (8, 26, 35, 38–43).

Other studies have found important differences between explicit and implicit attitudes (44). Explicit attitudes are attitudes that the individual himself is aware of, while implicit attitudes are those attitudes that one is not aware of, but can be measured indirectly through, for example, autonomous reactions. The most common approach to measuring explicit attitudes is self-reporting as these are attitudes that the subject is self-aware of. The SACS questionnaire measures only explicit attitudes. One of the studies included in this review had however studied both explicit and implicit attitudes (45). This is also a topic that should be investigated more thoroughly in future studies.

As shown in Table 3, the reporting of measurement properties except structural validity and internal consistency are so far almost non-existent in SACS studies. A major reason for this may be those analyses of factor structure and the internal consistency of scales and subscales are well established and fairly easy to do, while several of the other properties have been more defined as standards more recently and are less established. Some of these also require more demanding design, data collections and data analyses. The detailed and complicated criteria in the COSMIN rating instructions also makes it difficult to have simple descriptions in a table explaining what the ratings mean. Some criteria in the COSMIN Risk of Bias checklist have also been debated, like rating properties of an instrument based on whether the instrument confirms a hypothesis in a study, as it is impossible to assess in one study both the treatment effect and the responsiveness of an instrument (46).

A challenge in using the COSMIN method for this systematic review is also that the COSMIN taxonomy is originally developed for the assessment of patient-outcome measures. The SACS measures staff attitudes, which is another kind of phenomenon. In general, the scientific study of staff attitudes in mental health care seems to be scarce, and this field needs to be methodologically developed.

### Limitations and Recommendations for Future Research

Limitations in this review could be that the two pairs of assessors that assessed the studies could develop different consensus about how to interpret the findings in the papers. Another possible limitation could be that two of the authors were also involved in the development of the original SACS questionnaire and could possibly be biased. We sought to take this into account by collaborating in pairs with the authors not included in the development of the scale. All authors were also involved in the final quality assurance and interpretation of the findings. It is also a challenge and possible limitation in this review that the validation process differs between different studies and countries. Further the studies have used the scale differently. The SACS was developed and validated 15–20 years ago. While not formally investigated in this review; changes in attitudes to toward coercion in the society at large may indicate that a revision of the SACS item may be warranted.

## Conclusion

The SACS is, to our best of knowledge, the only questionnaire measuring staff's attitudes to the use of coercive interventions in mental health services. It is used widely, which demonstrates the need for such a tool. The widespread use also indicates that the tool is perceived as feasible and useful.

The assessment found evidence for adequate validity and internal consistency of the SACS. However, there were very limited support for other important measurement qualities such as reliability, criterion validity and measurement error.

Future research should focus on the stability of these attitudes, whether they are amendable by interventions and the relationship between staff attitudes to coercive interventions and the actual use of coercion. Another related topic is to more thoroughly investigate the relationship between staff's explicit and implicit attitudes toward use of coercion. Further future research could investigate formation of staff attitudes. Staff attitudes to the use of coercive practices may also influence the staff's willingness to engage in projects to reduce the use of coercive interventions. Another possible topic for research is to investigate barriers to engage in projects aimed at reducing use of coercive practices in mental health care.

Until future studies have evaluated more measurement properties of SACS, users of SACS must interpret the results based on the current knowledge of its properties.

## Data Availability Statement

The raw data supporting the conclusions of this article will be made available by the authors, without undue reservation.

## Author Contributions

All authors have been involved in the process of assessing the studies for inclusion, analyzing the results, writing article, contributed to the article, and approved the submitted version.

## Conflict of Interest

The authors declare that the research was conducted in the absence of any commercial or financial relationships that could be construed as a potential conflict of interest.

## Publisher's Note

All claims expressed in this article are solely those of the authors and do not necessarily represent those of their affiliated organizations, or those of the publisher, the editors and the reviewers. Any product that may be evaluated in this article, or claim that may be made by its manufacturer, is not guaranteed or endorsed by the publisher.
