# Supplementary material for: Measurement Properties of the Staff Attitude to Coercion Scale: A Systematic Review
Source: Front Psychiatry. 2022 Apr 28;13:744661. doi: 10.3389/fpsyt.2022.744661 (PMC9095955; doi:10.3389/fpsyt.2022.744661)
Supplement: Supplementary file 1 [file Data_Sheet_1.PDF]

Staff Attitude to Coercion Scale (SACS)

Search strategies

Date for search: 25-26 February 2021
Information specialist: Åse Marit Hammersbøen
Total number of hits from bibliographic databases & grey literature: **180**
Total number of hits after removing duplicates: **76**

Database: Ovid MEDLINE(R) ALL 1946 to February 24, 2021

#	Searches	Results
1	(staff attitude* adj5 coercion scale*).mp.	8
2	(staff* attitude* adj5 coerc*).mp.	9
3	(staff* adj5 attitude* adj5 coerc*).mp.	12
4	(sacs adj3 coerc*).mp.	5
5	1 or 2 or 3 or 4	12

Database: Embase 1974 to 2021 February 24

#	Searches	Results
1	(staff attitude* adj5 coercion scale*).mp.	8
2	(staff* attitude* adj5 coerc*).mp.	9
3	(staff* adj5 attitude* adj5 coerc*).mp.	12
4	(sacs adj3 coerc*).mp.	4
5	1 or 2 or 3 or 4	12

Database: APA PsycInfo 1806 to February Week 3 2021

#	Searches	Results
1	(staff attitude* adj5 coercion scale*).mp.	13
2	(staff* attitude* adj5 coerc*).mp.	14
3	(staff* adj5 attitude* adj5 coerc*).mp.	15
4	(sacs adj3 coerc*).mp.	4
5	1 or 2 or 3 or 4	15

Database: Cinahl

ID#	Search Terms	Results
S1	TX (staff attitude* N3 coercion scale*)	10
S2	TX (staff* attitude* N5 coerc*)	10
S3	TX (staff* N5 attitude* N5 coerc*)	13
S4	TX (sacs N3 coerc*)	8
S5	S1 OR S2 OR S3 OR S4	13

Database: **Web of Science** (Indexes=SCI-EXPANDED, SSCI, A&HCI, ESCI Timespan=1945-2021)

Set	Searches	Results
# 1	**TOPIC:** ("staff attitude*" NEAR/5 "coercion scale*")	8
# 2	**TOPIC:** ("staff* attitude*" NEAR/5 coerc*)	9
# 3	**TOPIC:** (staff* NEAR/5 attitude* NEAR/5 coerc*)	13
# 4	**TOPIC:** (sacs NEAR/3 coerc*)	5
# 5	#4 OR #3 OR #2 OR #1	**13**

Database: **Scopus**

Set	Searches	Results
1	TITLE-ABS-KEY ("staff attitude*" W/5 "coercion scale*")	9
2	TITLE-ABS-KEY ("staff* attitude*" W/5 coerc*)	10
3	TITLE-ABS-KEY (staff* W/5 attitude* W/5 coerc*)	15
4	TITLE-ABS-KEY (sacs W/3 coerc*)	6
5	#1 OR #2 OR #3 OR #4	**15**

Database: Cochrane Central Register of Controlled Trials (Issue 2 of 12, February 2021)
((staff* NEXT attitude*) AND coerc*):ti,ab,kw **0 hits**

Database: PubPsych
staff attitude coercion scale **8 hits**

Database: Google Scholar
"staff attitude to coercion" OR "staff attitude toward coercion" OR "staff attitude towards coercion" OR "staff attitude coercion" **66 hits**

Database: OpenGrey
"staff attitude to coercion" OR "staff attitude toward coercion" OR "staff attitude towards coercion" OR "staff attitude coercion" **0 hits**

Database: ProQuest Dissertations & Thesis
"staff attitude to coercion" OR "staff attitude toward coercion" OR "staff attitude towards coercion" OR "staff attitude coercion" **1 hits**

Database: Oria.no (Norwegian special libraries catalogue)
"staff attitude to coercion" OR "staff attitude toward coercion" OR "staff attitude towards coercion" OR "staff attitude coercion" **25 hits**

Citing articles search

Original article:
Husum, T. L., Finset, A., & Ruud, T. (2008). The Staff Attitude to Coercion Scale (SACS): reliability, validity and feasibility. *International Journal of Law & Psychiatry, 31*(5), 417-422.
https://doi.org/https://dx.doi.org/10.1016/j.ijlp.2008.08.002

Date for search: 25-26 February 2021

Databases and number of hits:

- Web of Science: 25
- Scopus: 28
- Google Scholar: 48

Total number of hits: **101**
Total number of hits after removing duplicates: **51**
